# Microvascular damage assessed by optical coherence tomography angiography for glaucoma diagnosis: a systematic review of the most discriminative regions

**DOI:** 10.1111/aos.14392

**Published:** 2020-03-16

**Authors:** Amerens Bekkers, Noor Borren, Vera Ederveen, Ella Fokkinga, Danilo Andrade De Jesus, Luisa Sánchez Brea, Stefan Klein, Theo van Walsum, João Barbosa‐Breda, Ingeborg Stalmans

**Affiliations:** ^1^ Biomedical Imaging Group Rotterdam Department of Radiology & Nuclear Medicine Erasmus MC Rotterdam The Netherlands; ^2^ Clinical Technology Delft University of Technology Delft The Netherlands; ^3^ Research Group Ophthalmology Department of Neurosciences KU Leuven Leuven Belgium; ^4^ Ophthalmology Department Centro Hospitalar e Universitário São João Porto Portugal; ^5^ Cardiovascular R&D Center Faculty of Medicine of the University of Porto Porto Portugal; ^6^ Department of Ophthalmology University Hospitals Leuven Leuven Belgium

**Keywords:** glaucoma, microvascular analysis, multilayer analysis, ocular blood flow, optical coherence tomography angiography, regions of interest, vascular damage

## Abstract

A growing number of studies have reported a link between vascular damage and glaucoma based on optical coherence tomography angiography (OCTA) imaging. This multitude of studies focused on different regions of interest (ROIs) which offers the possibility to draw conclusions on the most discriminative locations to diagnose glaucoma. The objective of this work was to review and analyse the discriminative capacity of vascular density, retrieved from different ROIs, on differentiating healthy subjects from glaucoma patients. PubMed was used to perform a systematic review on the analysis of glaucomatous vascular damage using OCTA. All studies up to 21 April 2019 were considered. The ROIs were analysed by region (macula, optic disc and peripapillary region), layer (superficial and deep capillary plexus, avascular, whole retina, choriocapillaris and choroid) and sector (according to the Garway–Heath map). The area under receiver operator characteristic curve (AUROC) and the statistical difference (p‐value) were used to report the importance of each ROI for diagnosing glaucoma. From 96 screened studies, 43 were eligible for this review. Overall, the peripapillary region showed to be the most discriminative region with the highest mean AUROC (0.80 ± 0.09). An improvement of the AUROC from this region is observed when a sectorial analysis is performed, with the highest AUROCs obtained at the inferior and superior sectors of the superficial capillary plexus in the peripapillary region (0.86 ± 0.03 and 0.87 ± 0.10, respectively). The presented work shows that glaucomatous vascular damage can be assessed using OCTA, and its added value as a complementary feature for glaucoma diagnosis depends on the region of interest. A sectorial analysis of the superficial layer at the peripapillary region is preferable for assessing glaucomatous vascular damage.

## Introduction

Glaucoma is the leading cause of irreversible blindness worldwide, with primary open‐angle glaucoma (POAG) as its most prevalent form (Van Melkebeke et al. 2018). Glaucoma is a multifactorial disease characterized by the loss of neural retinal ganglion cells. Classic theories attribute glaucomatous neuronal damage to mechanical trauma caused by elevated intraocular pressure (IOP) or to dysfunction of vascular perfusion and subsequent optic nerve ischaemia (Halpern & Grosskreutz 2002). Although elevated IOP remains the only confirmed modifiable risk factor for development and progression of glaucoma (Kass & Gordon 2000; Heijl et al. 2002; Jesus et al. 2017), differences in vascular parameters have been continuously reported between glaucoma and healthy individuals, at both ocular and systemic level (Barbosa‐Breda et al. 2019a, 2019b).

A number of techniques, such as fluorescein angiography, colour Doppler imaging, laser speckle flowgraphy and laser Doppler flowmetry, have been used in the evaluation of ocular and retinal blood perfusion (Michelson et al. 1996; Sugiyama et al. 2010; Stalmans et al. 2011; Spaide et al. 2015; Abegão Pinto et al. 2016; Barbosa‐Breda et al. 2019a, 2019b). The application of these modalities to glaucoma has contributed to a more comprehensive assessment of the role of vascular supply in the disease pathophysiology. With the introduction of new imaging modalities such as optical coherence tomography angiography (OCTA), standard OCT devices are now capable of analysing retinal blood flow, which is witnessed by extensive literature already published in ocular diseases (Fang et al. 2016; Koustenis et al. 2017). Due to the early stage of the technology, different strategies have been proposed and are currently used to retrieve the angiographic data from the OCT scans (e.g. the split‐spectrum amplitude‐decorrelation angiography (Jia et al. 2012), the OCT‐based microangiography (Zhang & Wang 2015), the OCTA ratio analysis (OCTARA) (Stanga et al. 2016) and the speckle variance OCTA (Xu et al. 2014)), which may lead to variability between results. Besides that, the quality of the OCTA scans may change significantly according to the acquisition parameters (e.g. number of images to be averaged) or artefacts such as eye movements. A number of studies have also developed and used different image processing algorithms to measure the vessel density from the OCTA images (e.g. percentage of vessel pixels in the respective region (Yip et al. 2019), mean intensity from the grayscale image (Jesus et al. 2019) or fractal analysis (Gadde et al. 2016)). Despite the differences that may exist between the OCTA imaging strategies and the algorithms to compute the vessel density, significantly lower vessel density and blood flow index in the macula (Akil et al. 2017; Chen et al. 2017; Chung et al. 2017; Alnawaiseh et al. 2018), optic disc (Bojikian et al. 2016; Chen et al. 2016; Cennamo et al. 2017; Chen et al. 2017; Chihara et al. 2017) and peripapillary region (Akil et al. 2017; Alnawaiseh et al. 2018; Lin et al. 2019) have been observed in glaucoma eyes in comparison with healthy ones. For all these regions, the diagnostic abilities increased with the severity of glaucoma (Chen et al. 2017; Chihara et al. 2017; Chung et al. 2017). Current results achieved with OCTA have presented it as a potential alternative or complementary technology for assisting glaucoma diagnosis. In comparison with the current imaging examinations used for the diagnosis and follow‐up, OCTA has shown to be less affected by the floor effect observed on structural OCT analysis and to require less patient cooperation than visual field testing (Van Melkebeke et al. 2018).

As in many medical imaging technologies at their early development stage, a number of approaches for estimating the microvascular density based on different regions of interest (ROIs) have been proposed. However, data reported in these approaches are often conflicting and/or arising from small‐scale studies, hindering the development of a general methodology to study glaucomatous vascular damage. Microvascular density measured from OCTA has shown to be device‐dependent, artefact‐dependent (e.g. eye motion, vitreous floaters, and media opacities) (Spaide et al. 2016; Sánchez Brea et al. 2019) and, more importantly, dependent on the imaged ROI. Since OCTA imaging is restricted to a narrow field of view, and the acquisition of a single image with good quality (i.e. no movement artefacts and good contrast) often requires a long exposure time (in patients known to have poor ocular surface and sometimes poor fixation capacities), it is important to ensure an efficient image acquisition, focusing first in the ROIs that yield more relevant information. Moreover, the distribution of the vascular glaucomatous damage among retinal, choriocapillaris and choroid layers is still under research. It is not clear yet whether the significant changes observed at the choriocapillaris and choroid are due to imaging artefacts or due to an actual disease mechanism (Sousa et al. 2019).

The aim of this systematic review was to contribute to the understanding of the role of vascular damage in glaucoma. To that end, the review focuses on the vascular density retrieved from the different ROIs that have been studied so far in the literature, reporting which ROIs have been found to be the most promising for studying glaucoma.

## Methods

This research adhered to the Preferred Items for Systematic Reviews and Meta‐analyses (PRISMA) guidelines.

### Study selection

A literature search was carried out in the PubMed database. The search query can be found in Appendix A. All studies that were published from the 1 January 2014 to the 21 April 2019 were included. The inclusion criteria for each study were as follows: (i) primary study, (ii) mention how the vessel density was computed, (iii) English language, (iv) conducted in humans, (v) investigate glaucomatous eyes in comparison with a healthy control group and (vi) reports at least: the area under the receiver operating characteristic curve (AUROC) or the statistical difference between the control and glaucoma groups. Four authors (A.B., N.B., V.E. and E.F.) screened all the titles and abstracts independently. A full‐text screening was carried out by two authors (N.B. and E.F.) independently. In case of disagreement, a third author (A.B. or V.E.) was consulted to reach consensus.

### Data collection

The extracted data included the following: study characteristics, AUROC values for different ROIs, microvascular density mean and standard deviation, and p‐values from the statistical comparison between healthy and glaucoma groups. If no statistical comparison or p‐values were provided, only the AUROC values were collected and *vice versa*.

For every study, the following characteristics were extracted: sample size including number of patients and eyes for each group, average age in years, and statistical difference (p‐value) between groups, glaucoma severity, OCT device brand and respective light‐source wavelength, cut‐off value for the signal strength index (SSI) (or similar image quality measure) used to exclude patients/eyes and field of view of the OCTA image.

**Fig. 1:**
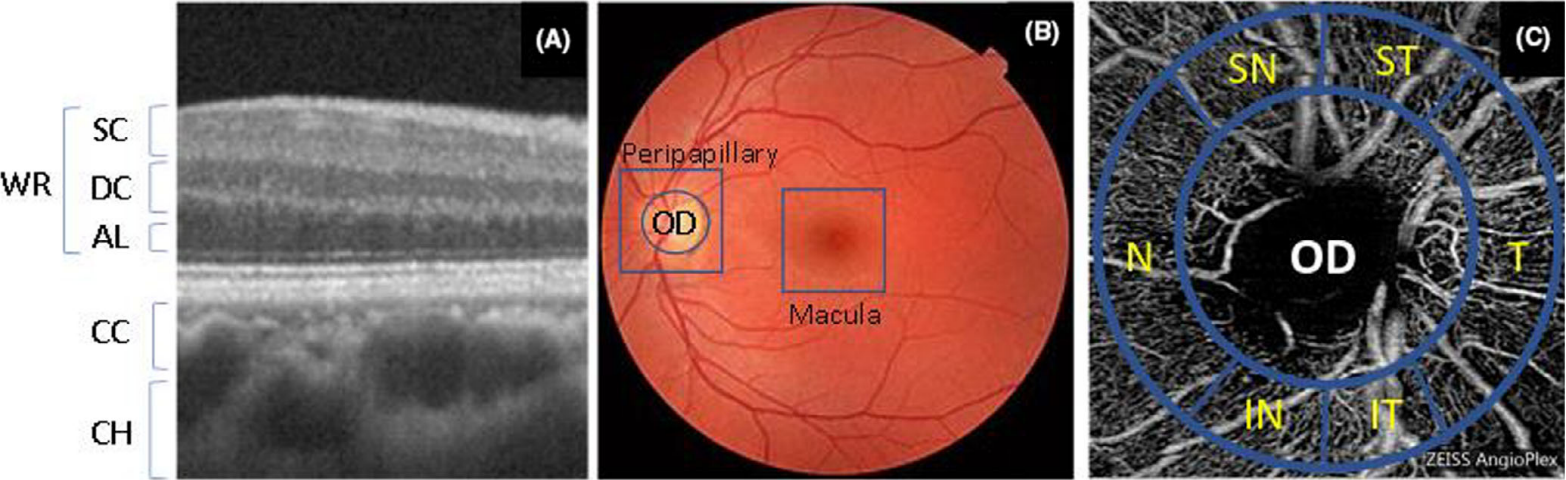
Regions of interest (ROIs) considered in this review for the analysis of the glaucomatous vascular damage. (A) Optical coherence tomography image showing the retinal superficial capillary plexus (SC), deep capillary plexus (DC), avascular layer (AL), whole retina (WR), choriocapillaris (CC) and choroid (CH). (B) Fundus image highlighting the macula, optic disc (OD) and peripapillary regions. (C) Optical coherence tomography angiography image with a circumpapillary representation of the Garway–Heath sectors: superonasal (SN), superotemporal (ST), temporal (T), inferotemporal (IT), inferonasal (IN) and nasal (N).

Data collection included the different layers: retina (including superficial, deep and avascular), choriocapillaris and choroid (Fig. 1A); the different regions: macula, optic disc (OD) and peripapillary or circumpapillary (when a circular band around the optic disc was considered instead of whole image) (Fig. 1B); and the sectors according to the Garway–Heath map (Garway‐Heath et al. 2000): superonasal (SN), superotemporal (ST), temporal (T), inferotemporal (IT), inferonasal (IN), nasal (N) and the inside disc (D) (Fig. 1C). The ‘whole retina’ was treated as a layer (Fig. 1A). Moreover, the ‘whole region’ (all the sectors combined) and the ‘fovea’ (centre of the macular region) were considered as sectors for the purpose of this review.

### Data analysis

The collected data were used to find which ROIs (region, layer and sector) have been studied and their discriminating power between glaucoma and healthy controls. Since the data analysis was oriented towards the clinical interpretation, the microvascular density was treated as a generic feature, not taking into account the different mathematical approaches used to estimate it.

The AUROC was considered as the most relevant metric for evaluating which ROIs are the most promising for studying glaucoma, since it provides the performance measurement for classification problem at various thresholds settings. The AUROC of all ROIs included in all reviewed studies was averaged to determine a threshold for selecting the studies that would undergo a qualitative assessment (described in section Qualitative assessment). The statistically significant differences (given as p‐values) were used to complement the information provided by the AUROCs and assess whether a ROI was relevant for differentiating glaucoma from healthy controls.

For those studies that did not report an AUROC, the decision to perform a qualitative assessment was based on the statistical comparison between the glaucoma and the healthy group. Hence, the ROIs that presented significant statistical differences (p‐value < 0.05) were also qualitatively evaluated.

### Qualitative assessment

There are several characteristics in a study that have been reported in the literature as potentially impacting the outcome of OCTA‐based glaucoma assessment and thus introducing bias. Thus, despite the high AUROC a method may have, it does not dismiss a careful qualitative analysis to identify these potential sources of bias. Hence, in this review, all the studies that reported an AUROC value above the threshold (mean AUROC values for all studied ROIs), and the ability to significantly differentiate glaucoma from healthy subjects, were qualitatively assessed. The qualitative assessment to measure the risk of bias was performed independently by two authors (A.B. and E.F.). Criteria were composed in cooperation with experienced ophthalmologists (J.B.B. and I.S.; Appendix B). The following six aspects, ordered by relevance, were considered:

1. *Age*. The age should not differ significantly between the glaucoma and the healthy groups. If there is an age difference between groups, an adjustment should be executed. Otherwise, the outcomes are considered as less reliable, because the microvascular density decreases with age (Lin et al. 2019).
2. *Eye*. Measurements obtained from both eyes of a subject are likely correlated. Hence, unless proper statistical methods are employed, there is a higher risk of bias if both eyes are included in the study.
3. *Type and severity of glaucoma*. Studies have a higher risk of bias when they report combined results of primary and secondary types of glaucoma, because of the difference in pathophysiology. Furthermore, the more severe the glaucoma, the more advanced the damage, not allowing to accurately infer the sensitivity of the studied feature. This means that a classification problem with high AUROC values for severe glaucoma may not be a good predictor for early diagnosis, even though they could still be good features for follow‐up.
4. *OCT specifications*. Different hardware specifications play a role in OCT image quality, especially in deeper layers such as the choriocapillaris and the choroid. Results from studies using different hardware should not be compared to each other but rather discussed separately.
5. *Image quality*. Studies that included images with SSI values (or similar quality measures) below the suggested inclusion value provided by the manufacturer are at higher risk of bias. Suggested values by manufacturers: for Angioplex^®^, include if >6 (out of 10); for AngioVue^®^, include if >45 (out of 100) (Spaide et al. 2016).
6. *Fovea‐disc axis correction*. If a sectorial analysis is performed, fovea‐disc axis correction should be executed for all the OCTA images to assure that the features are computed for the same ROIs between subjects (e.g. using a Panomap^®^ image; or any other reference of the relative position of the fovea and the optic disc (Mwanza et al. 2015; Jesus et al. 2019)).

## Results

### Study selection

**Fig. 2:**
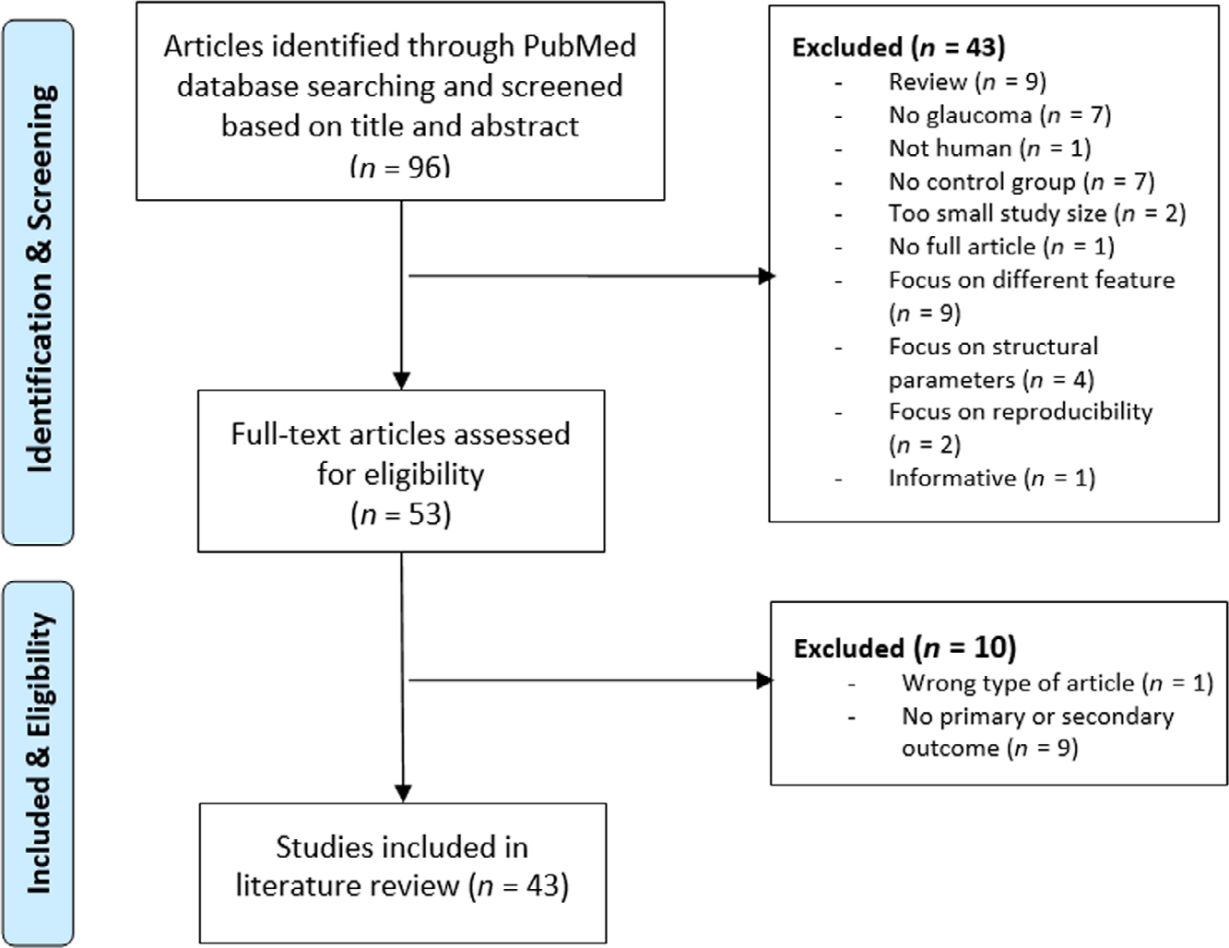
Flowchart of the study selection.

Ninety‐six studies were identified using the search query in Appendix A. From those, 53 studies were considered eligible after screening the titles and abstracts. Full‐text screening resulted in 43 studies that met all inclusion criteria and, hence, were eligible for the data analysis (Fig. 2). All the included studies provided a statistical analysis of the quantitative vascular evaluation for different ROIs. Twenty‐four studies provided AUROC as an outcome. The complete table with the characteristics of the reviewed studies can be found in Appendix B.

### AUROC analysis

**Fig. 3:**
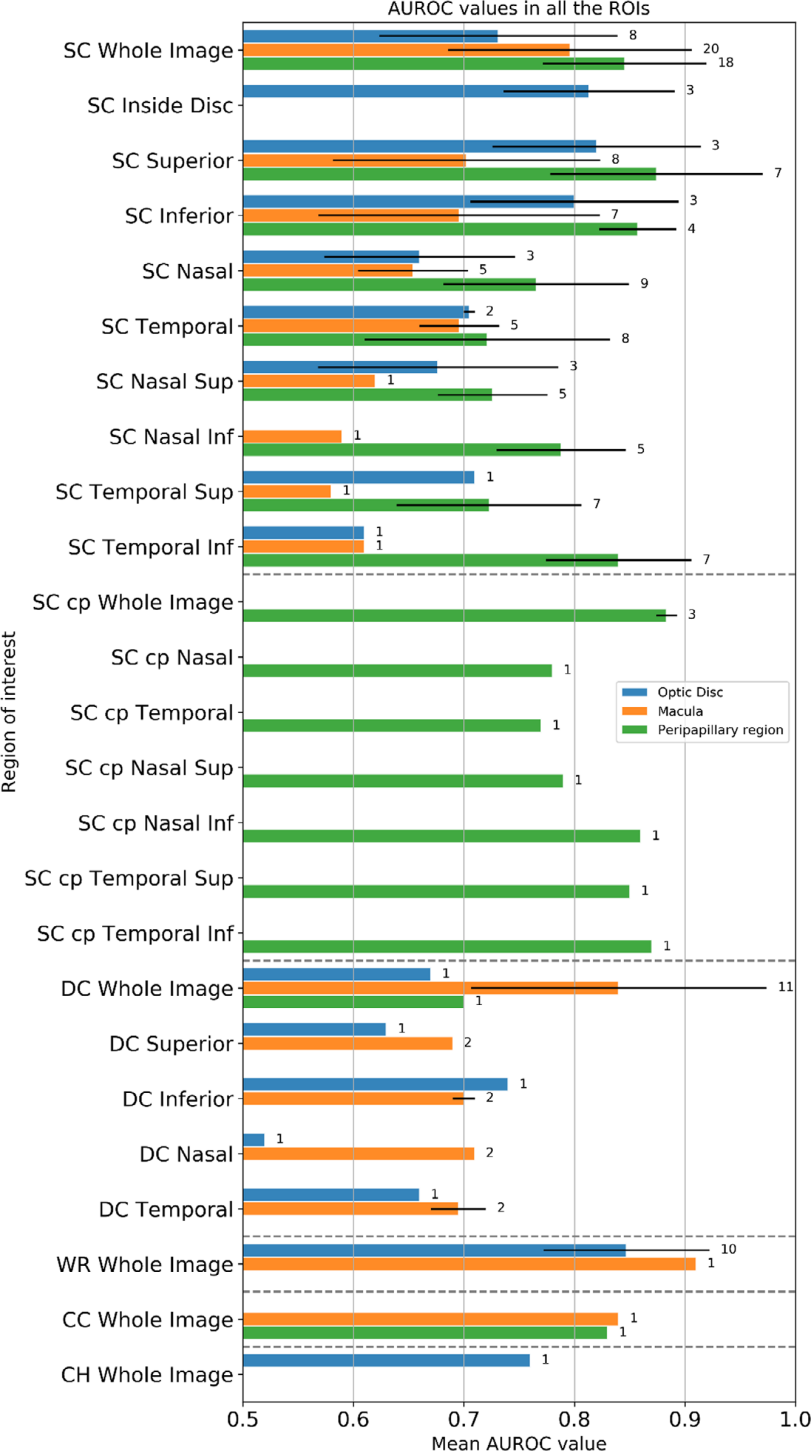
Mean AUROC and standard deviation value/number of observations for each ROI. AUROC = area under the receiver operating characteristic curve, CC = choriocapillaris, CH = choroid, cp = circumpapillary, DC = deep capillaris plexus, ROI = Regions of interest, SC = superficial capillaris, WR = whole retina.

The AUROCs presented in the reviewed studies are summarized in Table 1 organized per layer, region and sector. All studies calculated AUROC values based on the microvascular density, despite using different image processing techniques for intensity quantification or binarization. Although the macular region showed the highest AUROC values (considering all studies individually), when taking the mean of all ROIs, the peripapillary region had the highest AUROC of 0.80 ± 0.09, whereas the macula and the optic disc both had AUROC of 0.74 ± 0.12. The mean AUROC values for all studied ROIs are shown in Fig. 3. The average of all AUROC values in Table 1 is 0.77, which was set as the threshold for deciding whether a study or ROI should be further analysed in the qualitative assessment. All three regions (optic disc, macular and peripapillary) yielded values above this threshold, as shown in Table 1. Table 1 also shows that the avascular layer was not mentioned in any study, the choriocapillaris only in two studies (Yarmohammadi et al. 2016a; Alnawaiseh et al. 2018) and the choroid in one study (Yip et al. 2019). On the other hand, the whole retina, and the superficial and deep capillary plexuses have been investigated frequently.

**Table 1:** Area under the receiver operating characteristic curve (AUROC) for differentiating glaucoma eyes from healthy controls according to the region, layer and sector for all reviewed studies.

Sector	Region		
	Optic disc	Macula	Peripapillary region
1. Superficial capillary plexus			
Whole image/region	**0.92** (Yip et al. 2019) **0.78** (Shin et al. 2017) 0.77 (Rao et al. 2017c) 0.76 (Rao et al. 2017b) 0.75 (Alnawaiseh et al. 2018) 0.73 (Rao et al. 2017a) 0.57 (Chung et al. 2017) 0.57 (Chen et al. 2016)	**0.96** (Takusagawa et al. 2017) **0.94** (Rabiolo et al. 2018) **0.94** (Chung et al. 2017) **0.94** (Chen et al. 2017) **0.92** (Rabiolo et al. 2018) **0.84** (Yip et al. 2019) **0.84** (Rabiolo et al. 2018) **0.84** (Rabiolo et al. 2018) **0.82** (Rabiolo et al. 2018) **0.80** (Kurysheva et al. 2018) **0.79** (Rabiolo et al. 2018) **0.78** (Lommatzsch et al. 2018) 0.77 (Rabiolo et al. 2018) 0.75 (Kurysheva et al. 2018) 0.71 (Rao et al. 2017c) 0.70 (Triolo et al. 2017) 0.70 (Rao et al. 2017d) 0.69 (Alnawaiseh et al. 2018) 0.67 (Rao et al. 2017c)* 0.52 (Kwon et al. 2017)	**0.96** (Akil et al. 2017) **0.96** (Akil et al. 2017) **0.94** (Yarmohammadi et al. 2016a) **0.93** (Chen et al. 2017) **0.90** (Akil et al. 2017) **0.90** (Rolle et al. 2019) **0.88** (Triolo et al. 2017) **0.85** (Rao et al. 2017d) **0.85** (Rao et al. 2017b) **0.83** (Rao et al. 2017a) **0.82** (Akil et al. 2017) **0.81** (Chung et al. 2017) **0.80** (Cennamo et al. 2017) **0.80** (Kurysheva et al. 2018) **0.78** (Lommatzsch et al. 2018) 0.76 (Akil et al. 2017) 0.76 (Akil et al. 2017) 0.69 (Alnawaiseh et al. 2018)
Inside disc	**0.91** (Rao et al. 2017b) **0.81** (Alnawaiseh et al. 2018) 0.72 (Rolle et al. 2019) 0.60 (Kiyota et al. 2018)		
Superior	**0.95** (Geyman et al. 2017) **0.78** (Shin et al. 2017) 0.73 (Rao et al. 2017d)	**0.98** (Takusagawa et al. 2017) **0.79** (Kurysheva et al. 2018) 0.69 (Lommatzsch et al. 2018) 0.67 (Lommatzsch et al. 2018) 0.65 (Rao et al. 2017b) 0.65 (Rao et al. 2017d) 0.63 (Rao et al. 2017a) 0.56 (Triolo et al. 2017)	**1.00** (Akil et al. 2017) **0.98** (Akil et al. 2017) **0.95** (Chung et al. 2017) **0.86** (Chung et al. 2017) **0.82** (Rao et al. 2017d) 0.77 (Rao et al. 2017b) 0.74 (Triolo et al. 2017)
Inferior	**0.89** (Geyman et al. 2017) **0.84** (Shin et al. 2017) 0.67 (Rao et al. 2017d)	**0.98** (Takusagawa et al. 2017) 0.69 (Kurysheva et al. 2018) 0.69 (Rao et al. 2017d) 0.68 (Lommatzsch et al. 2018) 0.68 (Lommatzsch et al. 2018) 0.61 (Rao et al. 2017a) 0.54 (Triolo et al. 2017)	**0.89** (Chung et al. 2017) **0.88** (Rao et al. 2017d) **0.86** (Chung et al. 2017) **0.80** (Triolo et al. 2017)
Nasal	0.74 (Rao et al. 2017d) 0.70 (Rao et al. 2017a) 0.54 (Shin et al. 2017)	0.70 (Kurysheva et al. 2018) 0.68 (Lommatzsch et al. 2018) 0.68 (Lommatzsch et al. 2018) 0.65 (Rao et al. 2017d) 0.56 (Rao et al. 2017a)	**0.86** (Kurysheva et al. 2018) **0.85** (Chung et al. 2017) **0.84** (Rao et al. 2016) **0.82** (Chung et al. 2017) **0.78** (Rao et al. 2017d) 0.73 (Triolo et al. 2017) 0.72 (Rao et al. 2017b) 0.70 (Rao et al. 2017a) 0.59 (Rolle et al. 2019)
Temporal	0.71 (Shin et al. 2017) 0.70 (Rao et al. 2017d)	0.74 (Kurysheva et al. 2018) 0.72 (Lommatzsch et al. 2018) 0.71 (Lommatzsch et al. 2018) 0.67 (Rao et al. 2017d) 0.64 (Rao et al. 2017a)	**0.86** (Chung et al. 2017) **0.83** (Chung et al. 2017) **0.79** (Kurysheva et al. 2018) 0.75 (Rolle et al. 2019) 0.70 (Rao et al. 2017a) 0.68 (Triolo et al. 2017) 0.68 (Rao et al. 2017d) 0.48 (Rao et al. 2016)
Temporal superior	0.71 (Rao et al. 2017a)	0.58 (Triolo et al. 2017)	**0.83** (Rao et al. 2017b) **0.81** (Kurysheva et al. 2018) 0.76 (Rao et al. 2017a) 0.71 (Rao et al. 2017c)* 0.71 (Rao et al. 2017c) 0.68 (Rao et al. 2016) 0.56 (Rolle et al. 2019)
Nasal superior	**0.83** (Geyman et al. 2017) 0.61 (Rao et al. 2017a) 0.59 (Rao et al. 2017a)	0.62 (Triolo et al. 2017)	**0.78** (Kurysheva et al. 2018) **0.78** (Rao et al. 2017b) 0.72 (Rao et al. 2016) 0.70 (Rao et al. 2017a) 0.65 (Rolle et al. 2019)
Temporal inferior	0.61 (Rao et al. 2017a)	0.61 (Triolo et al. 2017)	**0.94** (Kurysheva et al. 2018) **0.89** (Rao et al. 2017a) **0.88** (Rao et al. 2016) **0.84** (Rao et al. 2017b) **0.83** (Rao et al. 2017c) 0.75 (Rao et al. 2017c)* 0.75 (Rolle et al. 2019)
Nasal inferior		0.59 (Triolo et al. 2017)	**0.88** (Kurysheva et al. 2018) **0.81** (Rao et al. 2017a) **0.78** (Rao et al. 2017b) 0.77 (Rao et al. 2016) 0.70 (Rolle et al. 2019)
Circumpapillary			
Whole image			**0.89** (Jesus et al. 2019) **0.89** (Chen et al. 2017) **0.87** (Kwon et al. 2017) 0.53 (Kiyota et al. 2018)
Nasal			**0.78** (Jesus et al. 2019)
Temporal			0.77 (Jesus et al. 2019)
Temporal superior			**0.85** (Jesus et al. 2019)
Nasal superior			**0.79** (Jesus et al. 2019)
Temporal inferior			**0.87** (Jesus et al. 2019)
Nasal inferior			**0.86** (Jesus et al. 2019)
2. Deep capillary plexus			
Whole image/region	0.67 (Shin et al. 2017)	**0.99** (Rabiolo et al. 2018) **0.99** (Rabiolo et al. 2018) **0.99** (Rabiolo et al. 2018) **0.97** (Rabiolo et al. 2018) **0.92** (Rabiolo et al. 2018) **0.86** (Yip et al. 2019) **0.79** (Rabiolo et al. 2018) 0.70 (Rabiolo et al. 2018) 0.70 (Lommatzsch et al. 2018) 0.70 (Alnawaiseh et al. 2018) 0.63 (Rao et al. 2017a)	0.70 (Lommatzsch et al. 2018)
Superior	0.63 (Shin et al. 2017)	0.69 (Lommatzsch et al. 2018) 0.69 (Lommatzsch et al. 2018)	
Inferior	0.74 (Shin et al. 2017)	0.71 (Lommatzsch et al. 2018) 0.69 (Lommatzsch et al. 2018)	
Nasal	0.52 (Shin et al. 2017)	0.71 (Lommatzsch et al. 2018) 0.71 (Lommatzsch et al. 2018)	
Temporal	0.66 (Shin et al. 2017)	0.72 (Lommatzsch et al. 2018) 0.67 (Lommatzsch et al. 2018)	
3. Whole retina			
Whole image/region	**0.96** (Yip et al. 2019) **0.93** (Akil et al. 2017) **0.91** (Rao et al. 2017c) **0.90** (Kurysheva et al. 2018) **0.86** (Akil et al. 2017) **0.84** (Kurysheva et al. 2018)^†^ **0.82** (Rao et al. 2017c)* 0.77 (Rao et al. 2017c)^†^ 0.74 (Alnawaiseh et al. 2018) 0.74 (Rao et al. 2017c)^†^	**0.91** (Takusagawa et al. 2017)	
4. Choriocapillaris			
Whole image/region		**0.84** (Alnawaiseh et al. 2018)	**0.83** (Yarmohammadi et al. 2016a)
5. Choroid			
Whole image/region	0.76 (Yip et al. 2019)		

The bold font highlights all numerical values above the selected threshold (AUROC> 0.77). No values were reported for the avascular layer. The whole image/region is defined as all sectors combined.

* With Disc Haemorrhage.

^†^ Inside disc.

#### Macular region

For the whole image of the macula in the superficial layer, 10 out of 20 values were reported above the threshold (Chung et al. 2017; Takusagawa et al. 2017; Kurysheva et al. 2018; Lommatzsch et al. 2018; Rabiolo et al. 2018; Yip et al. 2019), and 6 out of 11 values were above the threshold in the deep layer (Rabiolo et al. 2018; Yip et al. 2019). Only one value above the threshold was reported for the macula in the whole retina (Takusagawa et al. 2017) and choriocapillaris (Alnawaiseh et al. 2018).

#### Optic disc

The inside disc (Rao et al. 2017b; Alnawaiseh et al. 2018) and the inferior sector (Geyman et al. 2017; Shin et al. 2017) in the superficial layer were the ROIs with the highest AUROC, based on the reports of two studies. The whole region of the optic disc in the whole retina layer had 7 out of 10 values above the threshold (Akil et al. 2017; Rao et al. 2017c; Kurysheva et al. 2018; Yip et al. 2019).

#### Peripapillary and circumpapillary region

The whole region (Yarmohammadi et al. 2016a; Akil et al. 2017; Cennamo et al. 2017; Chung et al. 2017; Triolo et al. 2017; Rao et al. 2017a; Rao et al. 2017b; Rao et al. 2017d; Kurysheva et al. 2018; Lommatzsch et al. 2018; Rolle et al. 2019), superior (Akil et al. 2017; Chung et al. 2017; Rao et al. 2017d), inferior (Chung et al. 2017; Triolo et al. 2017; Rao et al. 2017d) and temporal inferior sectors (Rao et al. 2016; Shin et al. 2017; Rao et al. 2017a; Rao et al. 2017b; Kurysheva et al. 2018) in the superficial layer often presented AUROC values above the threshold. Also, for the whole region of the circumpapillary ROI (circular band in the peripapillary region) in the superficial layer, multiple values were reported above the threshold (Chen et al. 2017), (Kwon et al. 2017), (Jesus et al. 2019). Only one AUROC for the whole region in the choriocapillaris above the threshold (Yarmohammadi et al. 2016a) was reported.

### p‐value analysis

**Fig. 4:**
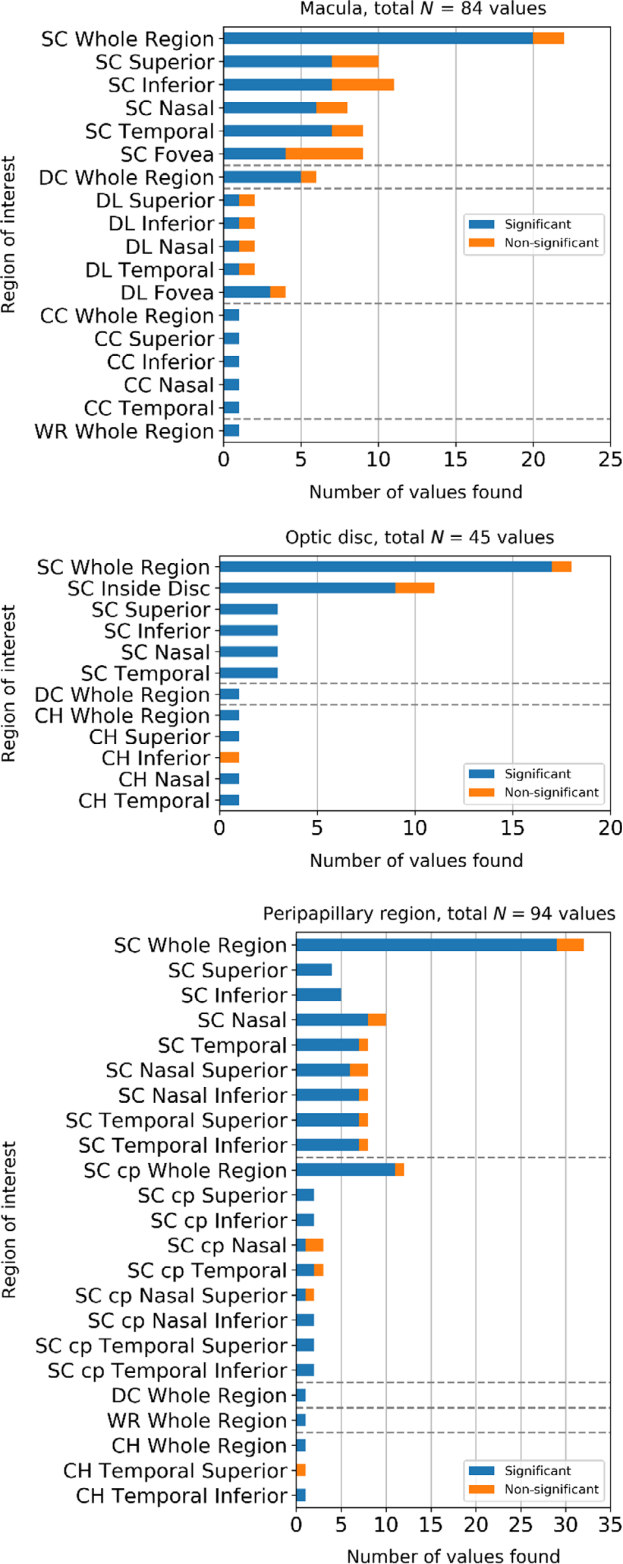
Number of studies with AUROC values> 0.77 for each ROI or that presented a significant (blue) and non‐significant (orange) statistical difference between healthy and glaucoma groups for the three regions; optic disc, macula and the peripapillary. AUROC = area under the receiver operating characteristic curve, CC = choriocapillaris, CH = choroid, cp = circumpapillary, DC = deep capillaris plexus, ROI = Regions of interest, SC = superficial capillaris, WR = whole retina.

The results for the vascular density differed greatly between and within ROIs, as shown in Appendix C. Nevertheless, a statistically significant difference between control and glaucoma groups was observed for all the analysed ROIs. The number of statistically significant differences is summarized in Fig. 4 (and detailed in Table C1 in Appendix C).

#### Macular region

The whole image of the macula in the superficial layer included 15 out of 17 significant values. Five out of six values reported for the whole image in the deep layer were significant. Only one value, however significant, was reported for the choriocapillaris and none for the choroid.

#### Optic disc

The inside disc sector in the superficial layer included nine significant values and only one non‐significant. The inferior segment in the superficial layer included only three values; however, all of them are significant. Only one out of 17 values reported a non‐significant difference for the whole image of the optic disc in the superficial layer. No values were reported for the whole image in the whole retina.

#### Peripapillary and circumpapillary region

The whole image in the superficial layer included 17 out of 19 significant values. The superior and inferior sectors of the peripapillary region in the superficial layer were not represented as much in the literature. However, all the studies that analysed these regions reported a significant difference between the groups (four and three values, respectively). Seven out of eight values for the temporal inferior sector in the superficial layer were significant. No values were reported for the whole region in the choriocapillaris. Five out of five values were reported as significant for the whole region of the circumpapillary ROI in the superficial layer. One value was reported for the temporal superior, temporal inferior and the nasal inferior sectors in the superficial layer, and all three of them were significant.

### Qualitative assessment

The bold font in Table 1 highlights the studies that provided one (or more) AUROC values above the threshold. The complete qualitative assessment was performed in the 22 studies that met the requirement of having an AUROC> 0.77 (Appendix D). From these study characteristics, it was possible to draw the following observations:

1. *Age.* Six studies (Rao et al. 2016; Yarmohammadi et al. 2016a; Geyman et al. 2017; Rao et al. 2017a; Rabiolo et al. 2018; Yip et al. 2019) reported a significant difference in age. However, all of them performed age correction.
2. *Eye.* Ten studies (Rao et al. 2016; Yarmohammadi et al. 2016a; Cennamo et al. 2017; Rao et al. 2017a; Rao et al. 2017b; Rao et al. 2017c; Rao et al. 2017d; Alnawaiseh et al. 2018; Rabiolo et al. 2018; Yip et al. 2019) included both eyes from the same subject. All of these studies except for Alnawaiseh et al. (Alnawaiseh et al. 2018), Cennamo et al. (Cennamo et al. 2017) and Yip et al. (Yip et al. 2019) mentioned to have performed a correction for this. Rolle et al. (Rolle et al. 2019) only mentioned the number of eyes and not the number of subjects included in the study.
3. *Type and glaucoma severity.* No patients with secondary glaucoma were included in any of the studies. All studies used a study population with different levels of glaucoma severity, however, Chen et al. (Chen et al. 2017), Jesus et al. (Jesus et al. 2019) and Yip et al. (Yip et al. 2019) used a patient group with a relatively low visual field mean deviation (MD) (respectively, −8.8 ± 6.2 dB, −7.8 ± 6.5 dB, and −11.07 ± 8.25 dB) when compared to the other studies with an average MD of −6.36 dB.
4. *OCT specifications.* All studies acquired the images with an OCT device with a light‐source wavelength of 840 nm, except Akil et al. (Akil et al. 2017), Rabiolo et al. (Rabiolo et al. 2018) and Triolo et al. (Triolo et al. 2017) which used an OCT system with a wavelength of 1040–1060 nm.
5. *Image quality*. Akil et al. (Akil et al. 2017) and Shin et al. (Shin et al. 2017) did not report whether they used a cut‐off value for exclusion due to image quality. However, they did report that 5 and 10 images, respectively, were not analysed because of poor OCTA image quality. Eight studies (Rao et al. 2016; Geyman et al. 2017; Rao et al. 2017a; Rao et al. 2017b; Rao et al. 2017d; Lommatzsch et al. 2018; Jesus et al. 2019; Yip et al. 2019) differed from the manufacturer's suggested cut‐off value. All these studies used a lower cut‐off value than the standard recommended value (Spaide et al. 2016).
6. *Fovea‐disc axis correction*. In none of the studies that performed a sectorial analysis, it was mentioned that a fovea‐disc axis correction was performed, except for Jesus et al. (Jesus et al. 2019).

## Discussion

This systematic review gives an insight into which ROIs have been studied so far in literature and which ones seem to contribute the most to an accurate diagnosis of glaucoma using microvascular density computed from OCTA. The ROIs in OCTA imaging were defined by three arguments: region of acquisition, layer and sector.

The region of acquisition (macula, optic disc or the peripapillary region) should be the first argument to be considered in OCTA imaging, since it is related to the ability to detect glaucomatous vascular damage. Although the highest AUROCs (considering all studies individually) were observed at the macula, the peripapillary region showed the highest AUROCs when averaging all values per region of acquisition. As mean AUROC is a more reliable indicator than its maximum, we may conclude that the peripapillary region is the most relevant for studying glaucomatous vascular damage.

The second argument to be considered is the layer. Overall, the highest AUROCs were obtained for the superficial layer. Nonetheless, the deeper layers presented in some cases similar classification values to the superficial layer. However, the limited number of studies that have covered these deeper layers does not allow to draw conclusions on their added value for the diagnosis. These layers have been avoided due to the difficulty to explain the physical meaning of the imaged content. As light travels deeper through retinal tissue, it becomes more susceptible to refraction and diffraction. Moreover, given the heterogeneity of retinal tissue, light reflection and absorption occur at different levels depending on the region of acquisition and respective refraction index. As a consequence, shadows are projected to deeper layers, creating what is known as projection artefacts. Therefore, and despite the significant differences observed at the choriocapillaris and the choroid, it is difficult to conclude whether these differences arise from the pathology itself or are a consequence of imaging artefacts. Further research needs to be done in order to understand to what extent the information imaged by OCTA at deeper layers is reliable.

The last argument, and the smallest area, is the sector. A sectorial analysis is not always performed in glaucomatous vascular studies. A number of studies have opted for analysing the retinal layers, mainly the superficial vascular plexus, without any sector discrimination. However, for those that performed sectorial analysis, it was shown that microvascular density is affected differently depending on the sector. Taking the most studied region of acquisition and layer as reference (the superficial layer of the peripapillary region), it can be concluded from this review that the inferior sector (AUROC = 0.86 ± 0.03) and the superior sector (AUROC = 0.87 ± 0.10) are the most promising at discriminating glaucoma. Moreover, Fig. 3 shows that a sectorial circumpapillary analysis (with a fixed distance from the optically hollow) seems to provide a better discrimination than a sectorial peripapillary configuration (which takes into account the entire scan). Such a difference may be explained by the reduced variability present in the circumpapillary region, a specific circular ROI with fixed dimensions around the optic disc.

Overall, looking at the number of studies that used OCTA information to infer glaucomatous vascular damage and the respective AUROCs, it can be concluded that the whole region at the superficial layer of the peripapillary ROI is the most accurate measurement for glaucoma assessment, which could be even further improved by a sectorial circumpapillary analysis. This result was somehow expected, since glaucoma is characterized by a loss of optic nerve axons, which traverse the retina superficially in an anatomical area included in the OCTA's superficial layer. Moreover, all the axons meet at the optic nerve which makes a circumpapillary analysis at the peripapillary ROI the best option to capture information from all of them at the same time. Macular scans are indeed relevant but can miss damage that falls outside the macular scan area (Van Melkebeke et al. 2018).

Nevertheless, a certain discrepancy and conflicting results have also been observed between sectors at different layers and regions of acquisition. Possible reasons for such a variability are related to the data and respective study design, and were qualitatively evaluated. Although no significant differences were observed in terms of age (except for one study which did not provide information (Rolle et al. 2019)), it was noted that three studies (Cennamo et al. 2017; Rao et al. 2017b; Alnawaiseh et al. 2018) used both eyes of the same subject, without mentioning any correction (Appendix D). Age and inclusion of both eyes can constitute a source of bias in the results, since the microvascular density decreases with age, and the data from both eyes are highly correlated. No secondary types of glaucoma were included in any of the reviewed studies. However, three of them (Chen et al. 2017; Jesus et al. 2019; Yip et al. 2019) used a glaucoma group with a relatively low visual field MD (−7.8 dB and lower) which may lead discrepancy between results. The comparison of different regions with data from groups with different severity groups may contribute to the misinterpretation of the data, as the more severe the glaucoma, the more advanced the damage is. Furthermore, three studies (Akil et al. 2017; Triolo et al. 2017; Rabiolo et al. 2018) used a 1040 nm OCT device and achieved a high diagnostic accuracy. All of these devices were Swept‐Source OCT (SSOCT) which could potentially indicate that a SSOCT may provide a better OCTA image quality and, consequently, may result in higher AUROCs. Further research is recommended to confirm the advantages of using SSOCT for OCTA imaging in assessing glaucomatous microvascular damage. A high risk of bias was identified in eight studies that included images with an image quality below the threshold suggested by the manufacturer (see Appendix D). Two other studies did not report which threshold was used. Only one study performed a fovea‐disc axis correction (Jesus et al. 2019). Due to eye motion or slight differences in position during image acquisition, OCTA images from different subjects might not match the same sectors at the same location. Therefore, sectorial analysis requires images to be previously corrected, for instance taking the fovea‐disc axis into account. This way all subjects will have the same reference point for the sectorial analysis.

Another reason for the current variability between studies is related to the method employed to extract vascular density. Although it is not the focus of this review, different image processing approaches can lead to different vascular interpretations within the same subject data. A popular method among the community is the OCTA image binarization based on thresholding techniques. The ratio of white or black pixels over a specific area is used to estimate the microvascular dropout. In general, the threshold is chosen based on an empirical analysis using an image processing programme such as ImageJ (Abràmoff et al. 2004). The separation of micro‐ from macrovasculature is another source of variability between studies. In some studies, the macrovasculature is segmented and removed from the region of acquisition. Other authors have opted for estimating vascular density based on all the information presented on the OCTA image. Macrovasculature is not expected to be affected by glaucoma, and it is a subject‐dependent anatomical feature. Thus, an analysis on image pixel intensity including macrovasculature is not desirable, as it may bias the results. Similarly, the optically hollow area inside the optic disc, as well as the foveal avascular zone (FAZ), is subject‐dependent. Therefore, it is desirable to segment and exclude these areas from the ROI before the microvascular density estimation is performed. Nevertheless, further research is needed for a better understanding of the variability between mathematical approaches and to understand which is the most appropriated for glaucoma diagnosis. Although a few research lines have already considered more complex procedures, such as fractal analysis (Gadde et al. 2016), replication studies are still needed to evaluate such advanced/complex methods.

The superior and inferior sectors of the superficial layer of the peripapillary region may be suitable for the diagnosis. However, the averaged AUROC reported in the reviewed articles is still lower than the values obtained with retinal nerve fibre layer thickness (measured through standard OCT imaging) and lower than the optic disc features (extracted from fundus imaging (Hemelings et al. 2020)), which usually result in AUROC values higher than 0.9. Nevertheless, recent studies have shown that vascular density assessed by OCTA seems to perform better than the gold standard biomarkers at discriminating advanced cases of glaucoma (Barbosa‐Breda et al. 2018; Van Melkebeke et al. 2018). Hence, follow‐up of (advanced) glaucoma using OCTA imaging may be a window of opportunity to establish OCTA as a common practice in the clinical environment. Thus, new studies will be required to infer which OCTA ROI is the best at glaucoma follow‐up.

### Conclusions

This review provides a comprehensive summary of the research on glaucomatous microvascular damage based on the analysis of different ROIs imaged with OCTA. The collected data show that the superficial layer in the peripapillary region is the most informative to infer vascular damage. Furthermore, at this location and layer, the inferior and superior sectors have been found as the most discriminative ROIs to study glaucomatous vascular damage with OCTA.

## Appendix A

Search query used in the PubMed database:

("Glaucoma*"[Mesh] OR “Glaucoma*”[tiab]) AND ("optical coherence tomography angiography"[tiab] OR "OCTA"[tiab] OR “OCT‐A”[tiab] OR “OCT angiography”[tiab] OR “optical coherence tomography based microangiography”[All Fields] OR “angio‐OCT”[tiab] OR “OCT‐angio”[tiab]) AND ("Glaucoma/diagnostic imaging"[Mesh] OR "Glaucoma/diagnosis"[Mesh] OR "Glaucoma/analysis"[Mesh] OR "Image Analysis"[tiab] OR "Image Processing"[tiab] OR "Image Enhancement"[Mesh] OR "Image Enhancement"[tiab] OR “Image processing, Computer Assisted”[Mesh] OR “Image processing, Computer Assisted”[tiab]OR "Computer‐Assisted Image Processing"[Mesh] OR "Computer‐Assisted Image Processing"[tiab]OR "Computer Assisted Image Processing"[Mesh] OR "Computer Assisted Image Processing"[tiab]OR "Image Reconstruction"[Mesh] OR "Image Reconstruction"[tiab] OR "Image Reconstructions"[Mesh] OR "Image Reconstructions"[tiab] OR "Reconstruction, Image"[Mesh] OR "Reconstruction, Image"[tiab]OR "Reconstructions, Image"[Mesh] OR "Reconstructions, Image"[tiab] OR"Image Analysis, Computer‐Assisted"[tiab]OR "Image Analysis, Computer Assisted"[Mesh] OR "Image Analysis, Computer Assisted"[tiab]OR "Computer‐Assisted Image Analysis"[Mesh] OR "Computer‐Assisted Image Analysis"[tiab]OR "Computer Assisted Image Analysis"[Mesh] OR "Computer Assisted Image Analysis"[tiab]OR "Analysis, Computer‐Assisted Image"[Mesh] OR "Analysis, Computer‐Assisted Image"[tiab]OR "Computer‐Assisted Image Analyses"[Mesh] OR "Computer‐Assisted Image Analyses"[tiab]OR "Image Analyses, Computer‐Assisted"[Mesh] OR "Image Analyses, Computer‐Assisted"[tiab] OR “Algorithm*”[tiab] OR “microvascular density”[tiab])

## Appendix B

**Table B1:** Characteristics of included studies. The publications in bold font were qualitatively assessed in this review.

Author	Number of patients/eyes	Eyes in control group (%)	Eyes in glaucoma group (%)	Age (years) (p‐value)	Type of glaucoma	OCT device	OCT light‐source wavelength (nm)	Image quality cut‐off value	Field of view (mm)
**Akil et al.** (2017)	56/56	16 (28.6)	20 mild POAG (35.7) 20 PPG (35.7)	62.2 versus 65.38 versus 63.13 p = 0.7	Mild POAG, PPG	DRI OCT, Triton, TOPCON	1050	NA	3 × 3
**Alnawaiseh et al.** (2018)	36/69	34 (49.3)	35 (50.7)	62 versus 63.09 p = 0.661	OAG	AngioVue	840	<50	3 × 3 pf 4.5 × 4.5 OD
Bojikian et al. (2016)	89/89	28 (31.5)	61 (68.5)	68.8 versus 66.2 versus 64.6 p = 0.38	POAG, NTG	Cirrus‐HD‐OCT‐5000, Zeiss AngioPlex	840	<6	6 × 6
**Cennamo et al.** (2017)	58/86	48 (55.8)	38 (44.2)	59.20 versus 65.05 p = 0.119	PPOAG	AngioVue	840	<50	7 × 7
Chen et al. (2016)	88/88	20 (22.7)	26 GS (29.5) 21 POAG (23.9) 21 NTG (23.9)	68.3 versus 68 versus 65.7 p = 0.51	GS, POAG, NTG	Cirrus‐HD‐OCT‐5000, Zeiss AngioPlex	840	<6	6 × 6
**Chen et al.** (2017)	53/53	27 (50.9)	26 (49.1)	57 versus 57 p = 0.84	POAG	AngioVue	840	<45	4.5 × 4.5 pp 6 × 6 macula
Chihara et al. (2017)	105/105	25 (23.8)	66 (76.2)	56.2 versus 60.4 p = 0.258	POAG	AngioVue	840	<40	4.5 × 4.5
Chung et al. (2017)	253/253	113 (44.7)	80 Early (31.6) 35 Moderate (13.8) 25 Severe (9.9)	49.8 versus 50.5 versus 52.9 versus 54.5 p = 0.133	Early, moderate, severe	AngioVue	840	<50	4.5 × 4.5
Fard et al. (2018)	78/125	80 (64.0)	45 (36.0)	48.4 versus 60.2 P = 0.08	POAG	AngioVue	840	<40	4.5 × 4.5
**Geyman et al.** (2017)	84/84	24 (28.6)	22 Mild (26.2) 20 Moderate (23.8) 18 Severe (21.4)	52 versus 66 versus 63 versus 62 p < 0.001	Mild, moderate, severe POAG	AngioVue	840	<40	4.5 × 4.5
**Jesus et al.** (2018)	122/122	40 (32.8)	82 (67.2)	63 versus 66 p‐value NA	PAOG, NTG	Cirrus‐HD‐OCT, Zeiss AngioPlex	840	<6	3 × 3
Jia y et al. (2014)	35/35	24 (68.6)	8 PG (22.9) 3 PPG (8.6)	52 versus 68 p = 0.000	PG, PPG	Ultrahigh speed swept‐source OCT imaging device	1050	NA	3 × 3
Kim et al. (2017)	22/44	9 (20.5)	13 (79.5)	35 versus 55.3 p < 0.001	PNTG	AngioVue	840	<48	4.5 × 4.5
Kiyota et al. (2018)	102/102	20 (19.6)	82 (80.4) ‐ 28 Mild (34.1) ‐ 25 Moderate (30.5) ‐ 29 Severe (35.4)	59 versus 60 versus 61 versus 61 p = 0.79	OAG, mild, moderate, severe	Swept‐source (SS‐OCT) Angio (Topcon)	1000	NA	4.5 × 4.5
Kromer et al. (2019)	51/51	21 (41.2)	30 (58.8)	70.3 versus 72.6 p = 0.298	POAG	SPECTRALIS, Heidelberg Engineering	870	NA	5 × 3.5
Kumar et al. (2016)	183/273	74 (27.1)	93 POAG (34.1) 70 PACG (25.6) Of which: ‐ 83 Early (30.4) ‐ 43 Moderate (15.8) ‐ 45 Severe (16.5) 28 PPG (10.3)	58.2 versus 57.04 versus 61.2 versus 62.6 versus 61.1 p < 0.001	PPG, early, moderate, severe	AngioVue	840	<40	4.5 × 4.5
**Kurysheva et al.** (2018)	125/125	35 (28.0)	48 Early POAG (38.4) 42 Moderate to severe POAG (33.6)	62.4 versus 53.7 versus 65.1 age‐matched	Early POAG, moderate to severe POAG	AngioVue	840	<50	4.5 × 4.5 OD 6 × 6 macula
**Kwon et al.** (2017)	125/125	45 (36.0)	45 PVFD (36.0) 35 CVFD (28.0)	49 versus 48 versus 51 p = 0.644	OAG, with PVFD, OAG with CVFD	Cirrus‐HD‐OCT, Zeiss AngioPlex	840	<8	3 × 3
**Lommatzsch et al.** (2018)	115/115	50 (43.5)	85 (56.5)	62 versus 61 p = 0.45	POAG, PEG, NTG, AAG	AngioVue	840	<40	6 × 6
**Lommatzsch et al.** (2018)	54/54	24 (44.4)	30 (55.6)	52 versus 62 p = 0.02	POAG, PEG, NTG AAG	Cirrus‐HD‐OCT, Zeiss AngioPlex	840	<8	6 × 6
Liu et al. (2015)	24/24	12 (50.0)	12 (50.0)	67 versus 70 age‐matched	PG, PPG	AngioVue	840	<40	3 × 3
Liu et al. (2017)	NA/43	27 (62.8)	16 (37.2)	55.8 versus 53.8 P = 0.46	OAG	AngioVue	840	<45	4.5 × 4.5 pp 6 × 6 macula
Mansoori et al. (2017)	76/76	52 (68.4)	24 (31.5)	49.89 versus 52.12 p = 0.57	POAG	AngioVue	840	<60	4.5 × 4.5
Poli et al. (2018)	36/52	15 (28.8)	15 PPG (28.8) 6 Mild (11.5) 16 Moderate to advanced (30.8)	39 versus 60 versus 63 versus 74 p = 0.002	PPG, POAG, mild moderate to advanced	AngioVue	840	<40	6 × 6 FAZ 4.5 × 4.5 OD
**Rabiolo et al.** (2018)	53/88	27 (30.7)	26 (69.3)	47.7 versus 60.5 p = 0.008	POAG	SS‐OCTA PLEX Elite 9000, Zeiss	1040–1060	<7	6 × 6
**Rao et al.** (2016)	92/142	78 (54.9)	64 (45.1)	58 versus 66 p = 0.01	POAG	AngioVue	840	<35	4.5 × 4.5 OD 3 × 3 macula
**Rao et al.** (2017a)	104/160	48 (30.0)	63 POAG (39.4) 49 PACG (30.7)	52 versus 65 versus 64 p < 0.001 and p = 0.002	POAG, PACG	AngioVue	840	<40	4.5 × 4.5
**Rao et al.** (2017c)	122/163	66 (40.5)	34 POAG with DH (20.9) 63 POAG without DH (38.7)	59.7 versus 65.6 versus 66.0 p = 0.93	POAG with DH, POAG without DH	AngioVue	840	<45	4.5 × 4.5 OD 3 × 3 macula
**Rao et al.** (2017d)	117/195	78 (40.0)	117 (60)	60.7 versus 62.8 p = 0.3	POAG	AngioVue	840	<35	4.5 × 4.5 OD 3 × 3 macula
**Rao et al.** (2017b)	117/173	77 (44.5)	31 PAC (17.9) 65 PACG (37.6)	60.7 versus 60.3 versus 62.0 p = 0.86 and p = 0.44	PAC, PACG	AngioVue	840	<35	4.5 × 4.5 OD 3 × 3 macula
**Rolle et al.** (2019)	NA/71	13 (18.3)	39 PPG (54.9) 19 POAG (26.8)	60.0 versus 63.4 versus 64.5 p = 0.24	PPG, POAG	AngioVue	840	<50	3 × 3
Scripsema et al. (2016)	92/92	26 (28.3)	40 POAG (43.5) 26 NTG (28.3)	63.72 versus 66.3 versus 67.92 p = 0.34	POAG, NTG	AngioVue	840	NA	3.5 × 3.5 4.5 × 4.5
**Shin et al.** (2017)	93/93	51 (54.8)	42 (45.2)	50.7 versus 51.8 p = 0.642	NTG	Cirrus‐HD‐OCT, Zeiss AngioPlex	840	NA	6 × 6
Nascimento E Silva et al. (2019)	46/46	16 (34.8)	30 (65.7)	67.9 versus 65.4 p = 0.37	POAG	DRI OCT, Triton, Topcon	1050	<48	3 × 3
**Takusagawa et al.** (2017)	60/60	30 (50.0)	30 (50.0)	65 versus 65 age‐matched	PG	AngioVue	840	<50	6 × 6
**Triolo et al.** (2017)	120/120	40 (30.0)	40 GS (30.0) 40 POAG (30.0)	60.9 versus 61.7 versus 63.6 p = 0.38	GS, POAG	SS‐OCTA PLEX Elite 9000, Zeiss	1040–1060	<7	6 × 6
Xu et al. (2018)	127/127	51 (40.2)	43 HTG (33.9) 33 NTG (26.0)	48.96 versus 50.91 versus 52.8 p = 0.467 and p> 0.99	HTG, NTG	AngioVue	840	<60	6 × 6 F 4.5 × 4.5 OD
**Yarmohammadi et al.** (2016)	164/261	23 (14.0)	37 GS (14.2) 104 OAG (39.8)	53.5 versus 68.2 versus 72.4 p < 0.001	GS, OAG	AngioVue	840	<48	4.5 × 4.5
(Yarmohammadi et al. 2016b)	152/152	26 (17.1)	52 GS (34.2) Mild 46 (30.3) Moderate and severe 28 (18.4)	55.6 versus 68.7 versus 72.9 versus 75.7 p < 0.001	GS, POAG, mild, moderate and severe	AngioVue	840	<48	4.5 × 4.5
Yarmohammadi et al. (2017)	86/86	28 (32.6)	58 (67.4)	69.91 versus 71.37 p = 0.190	OAG	AngioVue	840	<48	4.5 × 4.5 pp 3 × 3 macula
**Yip et al.** (2019)	53/90	58 (64.4)	15 POAG (16.7) 14 NTG (15.6) 1 juvenile OAG (1.1) 2 CAG (2.2)	51.17 versus 57.5 p = 0.051	POAG, NTG, CAG	AngioVue	840	<40	3 × 3
Zhu et al. (2018)	44/78	39 (50.0)	39 (50.0)	57.83 versus 59.93 p = 0.095	PACG	AngioVue	840	<40	4.5 × 4.5
Zivkovic et al. (2017)	51/51	21 (41.2)	30 (58.8)	69.5 versus 70.1 p = 0.70	NTG	Cirrus‐HD‐OCT, Zeiss AngioPlex	840	<6	3 × 3 FAZ

CVFD = central visual field defect, F = fovea, FAZ = foveal avascular zone, GS = glaucoma suspect, NA = not available, that is, not mentioned in the article, NTG = normal tension glaucoma, OAG = open‐angle glaucoma, OD = optic disc, P = perimetric, PACG = primary angle closure glaucoma, pf = parafoveal, POAG = primary open‐angle glaucoma, pp = peripapillary, PP = preperimetric, PVFD = peripheral visual field defect.

In the work published by Lommatzsch et al. (2018), two studies were performed. In each study, a different number of eyes were examined using one of two devices (Optovue or the Zeiss Cirrus).

## Appendix C

**Table C1:** Vessel density values (%) for healthy control group (bold) versus glaucoma patients according to the ocular layer, region and sector. Some studies provided multiple values for the same area due to comparison between approaches or different glaucoma types. The radial peripapillary capillary plexus is shown here as sublayer of the superficial capillary plexus.

Sector	Region		
	Optic Disc	Macular Region	Peripapillary region
1.a Radial peripapillary capillary plexus			
Whole image	**14.6** versus 5.3 (Yip et al. 2019) **51.8** versus 47.3 (Rabiolo et al., 2018) **49.3** versus 46.7 (Rabiolo et al. 2018) **49.5** versus 43.4 (Rabiolo et al. 2018) **48.1** versus 41.9 (Rabiolo et al. 2018) **49.7** versus 37.1 (Rabiolo et al. 2018) **40.8** versus 39.3 (Rabiolo et al. 2018) **55.42** versus 50.58 (Alnawaiseh et al. 2018)^†^ **53** versus 54 (Triolo et al. 2017) **53** versus 50 (Triolo et al. 2017) **55.8** versus 52.9 versus 43.1 (Kurysheva et al. 2018) **53.9** versus 45.2 (Rao et al. 2017d)* **58.8** versus 55.4 versus 48.6 versus 43.1 versus 36.5 (Kumar et al. 2016) **54.4** versus 50.4 (Rao et al. 2017a) **53.9** versus 45.1 (Rao et al. 2017b)* **58.09** versus 54.01 (Alnawaiseh et al. 2018)^†^ **86.6** versus 78.04 versus 70.1 (Akil et al. 2017)		**61.9** versus 54.4 (Rao et al. 2017d)* **47** versus 40 versus 46 (Chen et al. 2016) **63.62** versus 56.15 (Zhu et al. 2018) **48.03** versus 46.09 versus 45.34 versus 42.36 (Chung et al. 2017) **62** versus 55 (Rao et al. 2017a) **53.3** versus 43.8 (Chen et al. 2017) **40.7** versus 37.9 (Nascimento E Silva et al., 2019) **63.07** versus 59.99 (Alnawaiseh et al. 2018)* **60.8** versus 52.5 versus 52.7 (Rao et al. 2016)* **58.0** versus 56.9 versus 46.3 (Kurysheva et al. 2018) **61.9** versus 53.3 (Rao et al. 2017b)* **65.28** versus 60.34 (Rolle et al. 2019) **93** versus 80.55 (Liu et al. 2015) **49.8** versus 38.2 versus 42.1 (Chihara et al. 2017) **86.6** versus 86.83 versus 80.03 (Akil et al. 2017) **42.2** versus 35.1 versus 30.8 versus 28.3 (Geyman et al. 2017)^‡^ **42.3** versus 30.2 (Fard et al. 2018)^‡^ **53.01** versus 44.73 (Liu et al. 2017) **56.7** versus 51.6 (Yarmohammadi et al. 2016b) **56.7** versus 48.3 (Yarmohammadi et al. 2016b) **56.7** versus 41.7 (Yarmohammadi et al. 2016b) **41.32** versus 36.49 (Scripsema et al. 2016)^‡^ **41.32** versus 33.13 (Scripsema et al. 2016)^‡^ **41.88** versus 36.48 (Scripsema et al. 2016)^‡^ **41.88** versus 33.31 (Scripsema et al. 2016)^‡^ **55.70** versus 51.87 (Poli et al. 2018) **55.70** versus 46.21 (Poli et al. 2018) **55.70** versus 37.45 (Poli et al. 2018) not significant (Rao et al. 2017c; Poli et al. 2018; Rolle et al. 2019)
Inside Disc	**20.9** versus 11.0 (Yip et al. 2019) **55.45** versus 49.09 (Rolle et al. 2019) **48.1** versus 40.1 (Rao et al. 2017d)* **48.1** versus 46.5 (Rao et al. 2017b)* **46,78** versus 34.07 (Alnawaiseh et al. 2018)* **47.4** versus 40.2 (Rao et al. 2017a) **77** versus 68 versus 70 (Bojikian et al. 2016) **50.2** versus 45.3 versus 37.7 (Kurysheva et al. 2018) **37.9** versus 39.8 versus 29.8 versus 29.8 versus 26.7 (Kumar et al. 2016) Not significant (Nascimento E Silva et al., 2019; Rolle et al. 2019)		
Inferior	**23.1** versus 13.3 (Yip et al. 2019) **64.7** versus 53.9 (Rao et al. 2017d)* **48.7** versus 43.7 (Rao et al. 2017b)*		**64.7** versus 53.9 (Rao et al. 2017d)* **46** versus 37 versus 46 (Chen et al. 2016) **63.26** versus 59.72 (Zhu et al. 2018) **44.3** versus 36.7 versus 30.5 versus 24.7 (Geyman et al. 2017)^‡^ **43.9** versus 36.1 (Fard et al. 2018)^‡^
Superior	**21.4** versus 12.7 (Yip et al. 2019) **49.1** versus 40.8 (Rao et al. 2017d)* **49.1** versus 49.5 (Rao et al. 2017b)*		**63.6** versus 55.2 (Rao et al. 2017d)* **45** versus 39 versus 46 (Chen et al. 2016) **43.6** versus 35.2 versus 30.3 versus 26.3 (Geyman et al. 2017)^‡^ **43.7** versus 29.1 (Fard et al. 2018)^‡^
Nasal superior			**63.26** versus 59.72 (Zhu et al. 2018) **63.91** versus 60.71 versus 57.84 versus 49.42 (Chung et al. 2017) **62.9** versus 55.3 (Rao et al. 2017a) **59.1** versus 51.8 versus 53.0 (Rao et al. 2016)* **55.6** versus 54.7 versus 44.8 (Kurysheva et al. 2018) **59.4** versus 51.8 (Rao et al. 2017b)* not significant (Mansoori et al. 2017; Rao et al. 2017c)^‡^
Nasal inferior			**64.40** versus 55.85 (Zhu et al. 2018) **65.80** versus 60.94 versus 59.11 versus 45.07 (Chung et al. 2017) **63.4** versus 55.2 (Rao et al. 2017a) **62.0** versus 53.8 versus 53.1 (Rao et al. 2016)* **62.1** versus 59.2 versus 54.4 (Rao et al. 2017c) **55.8** versus 57.6 versus 45.0 (Kurysheva et al. 2018) **61.2** versus 54.3 (Rao et al. 2017b)* not significant (Mansoori et al. 2017)^‡^
Nasal	**17.3** versus 8.2 (Yip et al. 2019) **48.9** versus 41.2 (Rao et al. 2017d)* **48.9** versus 44.2 (Rao et al. 2017b)*		**59.5** versus 53.2 (Rao et al. 2017d)* **61.39** versus 54.61 (Zhu et al. 2018) **60.23** versus 59.21 versus 55.80 versus 48.77 (Chung et al. 2017) **59.7** versus 56 (Rao et al. 2017a) **58.0** versus 51.2 versus 51.9 (Rao et al. 2016)* **56.9** versus 54.8 versus 46.5 (Kurysheva et al. 2018) **42.8** versus 35.5 versus 33.2 versus 29.8 (Geyman et al. 2017)^‡^ **43.3** versus 32.2 (Fard et al. 2018)^‡^ not significant (Chen et al. 2016; Rao et al. 2017c)
Temporal superior			**66.25** versus 58.36 (Zhu et al. 2018) **68.82** versus 64.15 versus 56.55 versus 43.31 (Chung et al. 2017) **66.6** versus 59.4 (Rao et al. 2017a) **63.3** versus 56.5 versus 53.5 (Rao et al. 2016)* **60.4** versus 59.0 versus 49.2 (Kurysheva et al. 2018) **64.9** versus 56.9 (Rao et al. 2017b)* **20.2** versus 10.8 (Mansoori et al. 2017)^‡^ not significant (Rao et al. 2017c)
Temporal inferior			**66.95** versus 60.11 (Zhu et al. 2018) **68.52** versus 64.93 versus 61.04 versus 49.15 (Chung et al. 2017) **66** versus 55.5 (Rao et al. 2017a) **64.6** versus 49.7 versus 52.8 (Rao et al. 2016)* **60.5** versus 59.8 versus 44.4 (Kurysheva et al. 2018) **20.7** versus 13.4 (Mansoori et al. 2017)^‡^ **63.0** versus 51.8 (Rao et al. 2017b)* not significant (Rao et al. 2017c)
Temporal	**17.3** versus 11.0 (Yip et al. 2019) **44.3** versus 34.9 (Rao et al. 2017d)* **44.3** versus 36.9 (Rao et al. 2017b)*		**60.3** versus 56.5 (Rao et al. 2017d)* **68.83** versus 64.16 versus 62.13 versus 50.55 (Chung et al. 2017) **60.4** versus 57 (Rao et al. 2017a) **59.0** versus 55.0 versus 53.6 (Rao et al. 2016)* **59.4** versus 58 versus 47 (Kurysheva et al. 2018) **45.2** versus 41.3 versus 37.5 versus 36.2 (Geyman et al. 2017)^‡^ **44.6** versus 28.2 (Fard et al. 2018)^‡^ not significant (Rao et al. 2017c)
Circumpapillary Whole image			**34** versus 24 (Jesus et al. 2019)* **61.5** versus 53.3 (Chen et al. 2017) **51.9** versus 39.5 versus 34.4 (Kwon et al. 2017) **62.4** versus 59 versus 54.7 (Yarmohammadi et al. 2017) **64.2** versus 60.3 versus 55.1 (Yarmohammadi et al. 2016a) **65.1** versus 57.5 (Yarmohammadi et al. 2016b) **65.1** versus 49.6 (Yarmohammadi et al. 2016b) **42.45** versus 37.20 (Scripsema et al. 2016)^‡^ **42.45** versus 33.40 (Scripsema et al. 2016)^‡^ **42.99** versus 37.75 (Scripsema et al. 2016)^‡^ **42.99** versus 34.24 (Scripsema et al. 2016)^‡^ not significant (Yarmohammadi et al. 2016b)
Temporal			**30** versus 23 (Jesus et al. 2019)* **62.1** versus 55.3 (Chen et al. 2017) not significant (Liu et al. 2017)
Temporal inferior			**66.4** versus 46.2 (Chen et al. 2017) **67.29** versus 46.39 (Liu et al. 2017)
Temporal superior			**66.0** versus 55.9 (Chen et al. 2017) **64.78** versus 54.47 (Liu et al. 2017)
Superior			**34** versus 23 (Jesus et al. 2019)* **62.01** versus 55.37 (Liu et al. 2017)
Nasal			**30** versus 24 (Jesus et al. 2019)* not significant (Chen et al. 2017; Liu et al. 2017)
Nasal inferior			**63.6** versus 51.8 (Chen et al. 2017) **62.23** versus 54.66 (Liu et al. 2017)
Nasal superior			**59.2** versus 54.7 (Chen et al. 2017) not significant (Liu et al. 2017)
Inferior			**38** versus 23 (Jesus et al. 2019)* **64.76** versus 50.53 (Liu et al. 2017)
1. Superficial capillary plexus			
Whole image	Not significant (Nascimento E Silva et al., 2019)	**46.89** versus 42.31 (Lommatzsch et al. 2018) **44** versus 40 (Lommatzsch et al. 2018) **43.2** versus 38.5 (Chen et al. 2017) **46.21** versus 39.55 (Zivkovic et al. 2017) **52.51** versus 48.89 (Alnawaiseh et al. 2018)* **58.02** versus 52.37 (Rolle et al. 2019) **58.02** versus 49.09 (Rolle et al. 2019) **48.3** versus 44.7 (Rao et al. 2017a)* **49.8** versus 47.5 (Rao et al. 2017a) **11.5** versus 8.8 (Yip et al. 2019) **37.3** versus 31.8 (Kromer et al. 2019) **60.5** versus 47.2 (Takusagawa et al. 2017) **53.77** versus 51.63 (Alnawaiseh et al. 2018)* **50.7** versus 45.9 versus 42.2 (Kurysheva et al. 2018) **49.5** versus 46.9 (Rao et al. 2017d)* **53.8** versus 51.1 versus 48.3 (Yarmohammadi et al. 2017) **50.83** versus 46.99 (Zhu et al. 2018) **52.27** versus 50.83 versus 47.15 versus 45.46 (Chung et al. 2017) **49.5** versus 46.6 (Rao et al. 2017b)* **43.11** versus 38.46 (Liu et al. 2017) not significant (Chen et al. 2017; Triolo et al. 2017)	**56.6** versus 51.3 versus 46.2 (Yarmohammadi et al. 2016a)
Superior		**50.3** versus 47.5 (Rao et al. 2017d)* **51.60** versus 47.62 (Zhu et al. 2018) **49.2** versus 49.7 (Rao et al. 2017b)* **52.65** versus 51.60 versus 47.64 versus 45.74 (Chung et al. 2017) **50.8** versus 47.6 (Rao et al. 2017a) **51.60** versus 47.62 (Zhu et al. 2018) **53.2** versus 47.3 versus 47.4 (Kurysheva et al. 2018) not significant (Liu et al. 2017; Triolo et al. 2017; Kromer et al. 2019)	
Nasal		**48.3** versus 45.9 (Rao et al. 2017d)* **48.5** versus 47 (Rao et al. 2017a) **50.57** versus 45.99 (Zhu et al. 2018) **51.2** versus 46.8 versus 46.3 (Kurysheva et al. 2018) **52.24** versus 50.58 versus 47.87 versus 46.85 (Chung et al. 2017) **56.2** versus 43.58 (Kromer et al. 2019) not significant (Chen et al. 2017; Liu et al. 2017)	
Temporal		**49.6** versus 46.8 (Rao et al. 2017d)* **52.64** versus 51.13 versus 47.36 versus 46.05 (Chung et al. 2017) **59.7** versus 56.0 (Rao et al. 2017a) **49.8** versus 47.3 (Rao et al. 2017a) **51.95** versus 48.18 (Zhu et al. 2018) **52.4** versus 47.2 versus 46.1 (Kurysheva et al. 2018) **43.06** versus 39.71 (Liu et al. 2017) not significant (Chen et al. 2017; Kromer et al. 2019)	
Inferior		**51.0** versus 46.6 (Rao et al. 2017d)* **49.3** versus 48.9 (Rao et al. 2017b)* **51.38** versus 50.29 versus 46.17 versus 44.11 (Chung et al. 2017) **60.4** versus 57 (Rao et al. 2017a) **48.5** versus 47 (Rao et al. 2017a) **50.57** versus 45.99 (Zhu et al. 2018) **51.2** versus 46.8 versus 46.3 (Kurysheva et al. 2018) not significant (Chen et al. 2017; Liu et al. 2017; Triolo et al. 2017; Kromer et al. 2019)	
Fovea		**36.4** versus 32.1 versus 30.8 (Kurysheva et al. 2018) **53.61** versus 47.23 (Poli et al. 2018) **53.61** versus 41.98 (Poli et al. 2018) **53.61** versus 39.89 (Poli et al. 2018) not significant (Chen et al. 2017; Alnawaiseh et al. 2018; Lommatzsch et al. 2018; Poli et al. 2018; Kromer et al. 2019)*	
2. Deep capillary plexus			
Whole image	**44.1** versus 39.4 (Nascimento E Silva et al., 2019)	**53.28** versus 48.23 (Lommatzsch et al. 2018) **56.97** versus 54.95 (Alnawaiseh et al. 2018)* **23.3** versus 13.6 (Yip et al. 2019) **30.6** versus 30.2 (Kromer et al. 2019) **57.3** versus 52.2 versus 46.6 (Kurysheva et al. 2018) not significant (Takusagawa et al. 2017)	**59.93** versus 57.07 (Alnawaiseh et al. 2018)*
Superior		**62.1** versus 57 versus 54.1 (Kurysheva et al. 2018) not significant (Kromer et al. 2019)	
Temporal		**60** versus 55.8 versus 54.1 (Kurysheva et al. 2018) not significant (Kromer et al. 2019)	
Inferior		**59.3** versus 56 versus 52 (Kurysheva et al. 2018) not significant (Kromer et al. 2019)	
Nasal		**59.6** versus 56.3 versus 50.7 (Kurysheva et al. 2018) not significant (Kromer et al. 2019)	
Fovea		**33.3** versus 30.11 (Lommatzsch et al. 2018) **29.45** versus 31.75 (Alnawaiseh et al. 2018)* **35.4** versus 32.5 versus 26.3 (Kurysheva et al. 2018) not significant (Kromer et al. 2019)	
3. Choriocapillaris			
Whole image		**22.5** versus 15.3 (Yip et al. 2019)	
Inferior		**22.2** versus 15.8 (Yip et al. 2019)	
Superior		**22.8** versus 15.1 (Yip et al. 2019)	
Nasal		**22.9** versus 15.3 (Yip et al. 2019)	
Temporal		**24.3** versus 15.1 (Yip et al. 2019)	
4. Choroid			
Whole image	**22.8** versus 16.5 (Yip et al. 2019)		**63.2** versus 64.2 versus 57.9 (Kim et al. 2017)
Temporal inferior			**68.4** versus 64.7 versus 48.37 (Kim et al. 2017)
Temporal superior			not significant (Kim et al. 2017)
Superior	**26** versus 17.8 (Yip et al. 2019)		
Nasal	**23.6** versus 17.3 (Yip et al. 2019)		
Temporal	**19.2** versus 13.1 (Yip et al. 2019)		
Inferior	Not significant (Yip et al. 2019)		
5. Whole retina			
Whole image		**51.58** versus 47.27 versus 45.56 (Xu et al. 2018)^§^	**63.29** versus 55.57 versus 49.78 (Xu et al. 2018)^§^

All values are in mean, except when specified differently. Value were considered as significant at a cut‐off of <0.05

* Values given in median.

^†^ Whole enface.

^‡^ Large vessels extracted, capillary density.

^§^ Not further specified which layer was used.

## Appendix D

**Table D1:** Qualitative assessment for the studies with an AUROC value above the threshold of 0.77.

Author	Age	Eye	Types and glaucoma severity (VF MD, dB)	OCT specifications	Image Quality cut‐off	Fovea‐Disc axis
Akil et al. (2017)	No	No	POAG: 2.8 ± 1.9 PPG: 1.65 ± 2.25	**1050**	NA*	**Yes**
Alnawaiseh et al. (2018)	No	**Yes**	−2.74 (−5.17, −1.25)^†^	840	<50	No, NA
Cennamo et al. (2017)	No	**Yes**	0.15 ± 1.17	840	<50	No, NA
Chen et al. (2017)	No	No	**–8.8 ± 6.2**	840	<45	**Yes**
Chung et al. (2017)	No	No	Early: −2.42 ± 1.69 Moderate: −8.35 ± 1.77 Severe: −20.12 ± 5.31	840	<50	**Yes**
Geyman et al. (2017)	Yes, corrected	No	Mild: −3.2 ± 1.7 Moderate: −8.0 ± 2.0 Severe: −21.4 ± 7.1	840	**<40**	**Yes**
Jesus et al. (2019)	No	No	**–7.8 ± 6.5**	840	**<6** ^‡^	No
Kurysheva et al. (2018)	No	No	Early POAG: −2.1 ± 3.4 Moderate/Severe POAG: −11.8 ± 6.1	840	<50	**Yes**
Kwon et al. (2017)	No	No	PVFD: −4 ± 4 CVFD: −3.2 ± 2.2	840	<8^‡^	No, NA
Lommatsch et al. (2018)	Yes, corrected	no	AngioVue group: − 4.8 ± 6.31 AngioPlex group: − 4.63 ± 5.7	840	**<40**	**Yes**
Rabiolo et al. (2018)	Yes, corrected	yes, corrected	NA	**1040–1060**	<7^§^	No, NA
Rao et al. (2016)	Yes, corrected	yes, corrected	–5.3 (−9.6, −3.1)^†^	840	**<35**	**Yes**
Rao et al. (2017a)	Yes, corrected	yes, corrected	POAG: −6.3 (−13.5, −3.1)^†^ PACG: −9.2 (−16.0, −3.3)^†^	840	**<40**	**Yes**
Rao et al. (2017d)	NO	Yes, corrected	–6.3 (−12.5, −3.5)^†^	840	**<35**	**Yes**
Rao et al. (2017b)	No	Yes, corrected	PAC: −1.9 (−3.6, −0.8)^†^ PACG: −8.2 (−16.0, −4.0)^†^	840	**<35**	**Yes**
Rao et al. (2017c)	No	Yes, corrected	POAG with DH: −3.7 (−6.3, −2.5)^†^ POAG without DH: −3.8 (−7.5, −2.8)^†^	840	<45	**Yes**
Rolle et al. (2019)	No	**Number of patients NA**	PPG: −0.96 ± 1.15 POAG: −4.63 ± 3.53	840	<50	**Yes**
Shin et al. (2017)	No	No	–5.88 ± 6.01	840	**NA** ^‡^	**Yes**
Takusagawa et al. (2017)	No	No	–5.32 ± 3.50	840	<50	No, NA
Triolo et al. (2017)	No	No	–5.45 ± 2.27	**1040–1060**	<7^§^	**Yes**
Yarmohammadi et al. (2016a,b)	Yes, corrected	Yes, corrected	–3.9 (−8.8, −1.8)^†^	840	<48	No, NA
Yip et al. (2019)	Yes, corrected	**Yes**	**–11.07 ± 8.25**	840	**<40**	

If a risk of bias for age or eye existed, yes was filled in. All values for visual field mean deviation are given in mean ± SD, unless indicated otherwise. All images were acquired using AngioVue OCTA device, unless indicated otherwise. Corrections for age and/or the use of both eyes for one subject are presented in the table when executed (corrected). If no sector analysis was executed in the study, a fovea‐disc axis correction was considered as unnecessary or not applicable (NA). Study characteristics that may bias the results are given in bold.

CFVD = central visual field defect, DH = disc haemorrhage, MD = mean deviation, PACG = primary angle closure glaucoma, PFVD = peripheral visual field defect, POAG = primary open‐angle glaucoma, PPG = preperimetric glaucoma, VF = visual field.

* DRI OTC, Triton, Topcon.

^†^ Values in median (IQR).

^‡^ Cirrus‐HD‐OCT‐5000, Zeiss Angioplex.

^§^ SS‐OCTA PLEX Elite 9000, Zeiss.
